# Crosstalk between 5-Aminolevulinic Acid and Abscisic Acid Adjusted Leaf Iron Accumulation and Chlorophyll Synthesis to Enhance the Cold Tolerance in *Solanum lycopersicum* Seedlings

**DOI:** 10.3390/ijms241310781

**Published:** 2023-06-28

**Authors:** Zhen Kang, Yong Zhang, Xiongchun Cai, Zhengda Zhang, Zijian Xu, Xiangguang Meng, Xiaojing Li, Xiaohui Hu

**Affiliations:** 1 College of Horticulture, Northwest A&F University, Yangling 712100, China; 2 Key Laboratory of Protected Horticultural Engineering in Northwest, Ministry of Agriculture, Yangling 712100, China; 3 Shaanxi Protected Agriculture Research Centre, Yangling 712100, China

**Keywords:** ABA, chlorophyll synthesis, low-temperature stress, *Solanum lycopersicum*, tomato seedling, 5-aminolevulinic acid

## Abstract

Previous studies found that 5-aminolevulinic acid (ALA) and abscisic acid (ABA) can mitigate damage from adversity by enhancing photosynthesis. However, it is not clear whether they have positive effects on iron utilization and chlorophyll synthesis of tomato seedlings under low-temperature stress. To investigate the possible functional relationship between ABA and ALA and elucidate the possible mechanisms of action of ALA to alleviate low-temperature stress in tomato seedlings, this experiment analyzed the effects of ALA and ABA on chlorophyll synthesis in tomato seedling leaves sprayed with exogenous of ALA (25 mg·L^−1^) or ABA (100 µM) under low-temperature stress (8–18 °C/8–12 °C, day/night). The results show that exogenous ALA increased the Fv/Fm of tomato leaves by 5.31% and increased the accumulation of iron and chlorophyll by 101.15% and 15.18%, respectively, compared to the low-temperature treatment alone, and tomato resistance of low-temperature stress was enhanced. Meanwhile, exogenous application of ALA increased the ABA content by 39.43%, and subsequent application of exogenous ABA revealed that tomato seedlings showed similar effects to exogenous ALA under low-temperature stress, with increased accumulation of iron and chlorophyll in tomato seedlings, which eventually increased the maximum photochemical efficiency of PS II. Under low-temperature stress, application of exogenous ABA significantly reduced ALA content, but the expression of key enzyme genes (*PPGD*, *HEMB1*, *HEME1*, and *HEMF1*), precursors of chlorophyll synthesis by ALA, was significantly elevated, presumably because the increased activity of these enzymes after external application of ABA accelerated ALA consumption. In conclusion, ABA may crosstalk with ALA to improve the photochemical efficiency and low temperature resistance of tomatoes by regulating chlorophyll synthesis and iron accumulation.

## 1. Introduction

Tomato (*Solanum lycopersicum*) is an important horticultural crop that is widely grown around the world and plays an important role in international trade [1]. As its main product organ, fruit provides rich nutrients for people. However, tomatoes are sensitive to low temperatures because they originated in tropical and subtropical regions [2]. Low temperature inflicts multiple changes in tomato plants, including chlorophyll synthesis, nutrient utilization, and reproductive growth, which is one of the important environmental limiting factors affecting plant growth, development, and geographical distribution [2,3].

In winter and spring in China, low-temperature stress (8–18 °C) often affects the normal growth of tomatoes grown in a greenhouse [1,2]. Low-temperature stress leads to the disruption of the balance of reactive oxygen species (ROS) in plants, resulting in a large accumulation of ROS [3]. The accumulation of ROS causes severe membrane lipid peroxidation and increased malondialdehyde (MDA) levels in plants [4]. The disruption of cell membranes leads to disruption of cellular osmotic balance and cytoplasmic efflux, and low-temperature stress can also disrupt organelle integrity and reduce cell membrane fluidity [5]. At the same time, low-temperature stress reduces the chlorophyll content and affects various physiological and biochemical processes of plants, ultimately leading to a decline in quality and yield [6,7,8]. The inhibitory effects of low temperatures on plant growth and development are closely related to the decrease in the content of mineral elements in leaves [9]. Iron can participate in the synthesis of chlorophyll, promote photosynthesis, and play an important role in the life activities of plants [10]. Iron is a component of the enzymes required for chlorophyll synthesis in plants such as porphobilinogen synthase and Mg-protoporphyrin Ⅸmethyltransferase, which must be present in chlorophyll synthesis [11]. Iron deficiency directly leads to the degradation of chloroplasts in the leaves of plants, affects the synthesis of the chlorophyll protein complex, and ultimately reduces the content of chlorophyll [12]. MtZIP3 is a zinc transporter with an iron transport activity that can respond to iron deficiency stress, *ZIP1* and *ZIP3* play an important role in the process of iron utilization by plants [13,14].

As a non-protein amino acid, 5-aminolevulinic acid (ALA) is widely present in animals, plants, fungi, and bacteria [1]. It is the precursor to porphyrins, which are, in turn, the precursors to plant pigments [15,16]. As one of the main pigments of plants, chlorophyll plays a vital role in plant growth and development. Very low concentrations (below 50 nmol/kg) of 5-aminolevulinic acid in plants can have a tight control on chlorophyll biochemical synthesis. The blocking of ALA synthesis leads to blockage of chlorophyll synthesis, which in turn causes the yellowing of plant leaves [17]. Exogenous ALA is easily absorbed by plants [18], and it can promote the content of chlorophyll synthesis precursors in plants, thereby increasing the chlorophyll content and the net photosynthetic rate of plant leaves under various stresses, including low temperatures [19]. In addition, ALA cannot only affect enzymatic and non-enzymatic antioxidants to remove active oxygen at low temperatures and protect the structure and function of cell membranes, but also regulate endogenous hormone levels and nutrient accumulation, thereby effectively enhancing the plant’s ability to resist stress [2,20,21,22,23].

The hormone abscisic acid (ABA) participates in a wide range of physiological and biochemical processes in plants, and it plays an important role in plant growth and development in various adverse environments [24]. Studies show that spraying exogenous ABA can increase the net photosynthetic rate and water use efficiency of plant leaves and the chlorophyll content of various crops, thereby alleviating adversity stress [25,26]. Under adversity stress, plants improve their stress resistance by synthesizing ABA [27].

Both ALA and ABA can mitigate plant damage from adversity by enhancing photosynthesis, and an increasing number of studies show that the two have similar roles in plant resistance to stress [22,23,24,25,26]. Is there a synergistic effect of ABA and ALA in low-temperature stress? At the same time, iron and chlorophyll are essential substances involved in photosynthetic processes in plants. Do ALA and ABA have any effect on iron utilization and chlorophyll synthesis in tomato seedlings under low-temperature stress? How do the two interact with each other? Additionally, how do they affect abiotic stresses? What are the genes that play a role in the joint? These questions prompted us to go deeper into the study. It is necessary to explore the functional relationship between them in the process of chlorophyll synthesis in plants. In this study, exploring the possible functional link between ALA and ABA and the effect of ALA on chlorophyll utilization by ABA in tomato seedlings under low temperature conditions and studying the accumulation of ALA and ABA in alleviating low-temperature stress can help us further understand the mechanisms of tomato stress resistance and provide new ideas for alleviating low-temperature stress in facility tomatoes. Additionally, studying the mechanism of the ABA–ALA interaction can provide new insights to explore the response mechanism of plants to abiotic stresses.

## 2. Results

### 2.1. Exogenous ALA Improved the Low-Temperature Stress Resistance of Tomato Seedlings

**Figure 1:**
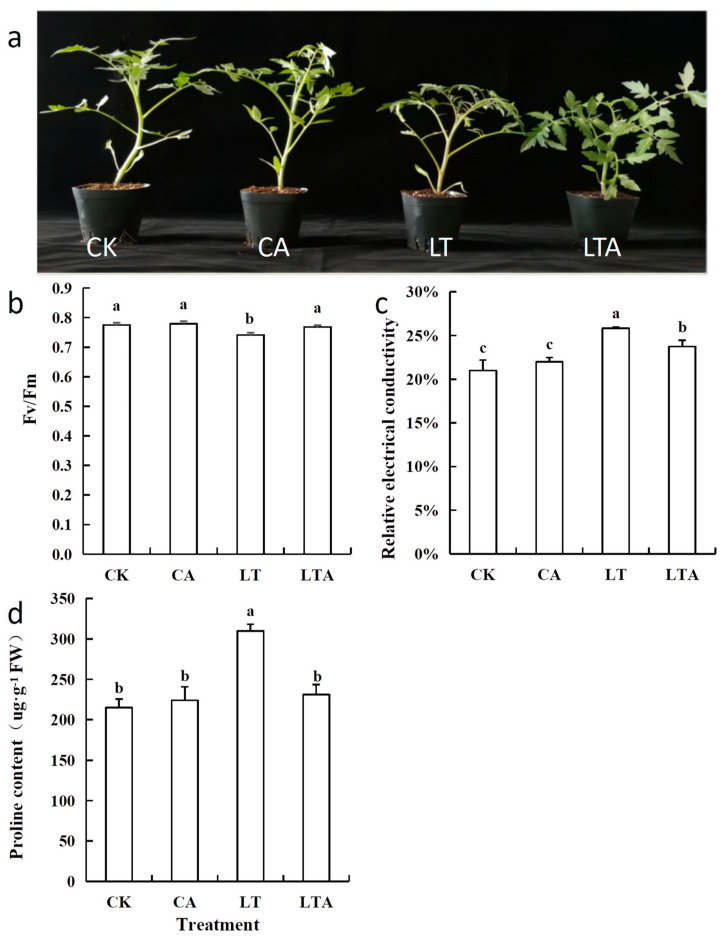
Exogenous ALA improved low-temperature resistance of tomato seedlings. Some of the tomato seedlings with five true leaves were grown at normal temperature and pre-sprayed with distilled water or ALA. Some of the tomato seedlings were grown at low temperature and pre-sprayed with distilled water or ALA. After nine days of low-temperature treatment, (**a**) plant growth status, (**b**) Fv/Fm, (**c**) relative electrical conductivity, and (**d**) proline content were measured. Values represent averages of three independent measurements, and error bars represent standard errors. Different letters above the columns indicate significant difference according to the Tukey test (*p* < 0.05).

To investigate the effect of ALA on tomato seedlings at low temperature, the growth of tomato seedlings with or without spraying exogenous ALA before low temperature was observed, and relevant physiological indices were measured. Tomato seedlings sprayed with ALA grew significantly better than CK at low temperature, while there was no significant positive effect at normal temperature (Figure 1a, Figure S4). Low-temperature stress resulted in a significant decrease in Fv/Fm by 6.36%, and caused the relative conductivity; proline content increased by 25.58% and 44.19%, respectively, of tomato seedling leaves compared to CK. In contrast, spraying exogenous ALA (LTA) greatly attenuated the changes in these indicators (Figure 1b–d). The results show a significant increase or decrease in LTA compared to LT with 5.31%, 6.20%, and 25.48%, respectively. Finally, the growth of tomato seedlings was significantly promoted (Figure 1a, Figure S4) at low temperature. These results show that exogenous ALA can significantly improve the low-temperature resistance of tomato seedlings.

### 2.2. Exogenous ALA Increased Contents of Iron and Chlorophyll in Tomato Seedlings Leaves under Low Temperature

**Figure 2:**
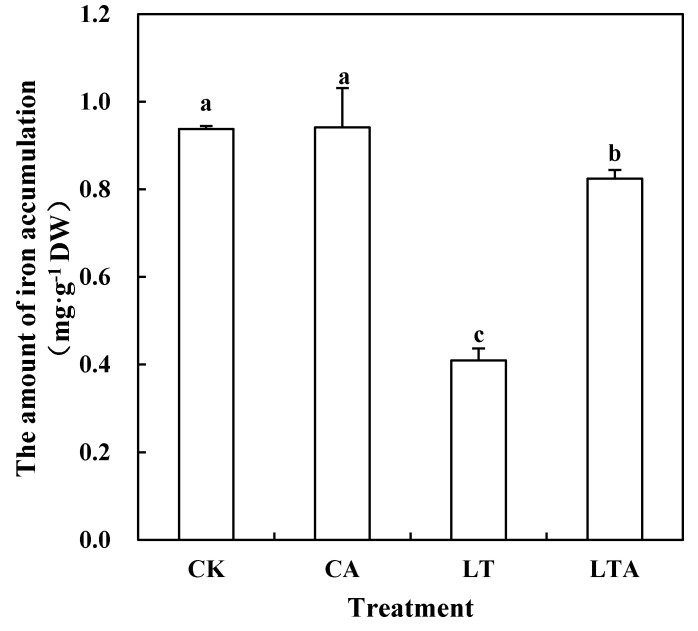
Effects of exogenous ALA on iron accumulation in tomato seedling leaves. Iron accumulation was measured at different temperatures for nine days. Some of the tomato seedlings with five true leaves were grown at normal temperature and pre-sprayed with distilled water or ALA. Some of the tomato seedlings were grown at low temperature and pre-sprayed with distilled water or ALA. Values represent averages of three independent measurements, and error bars represent standard errors. Different letters above the columns indicate significant difference according to the Tukey test (*p* < 0.05).

Previous studies showed that ALA can promote the synthesis of chlorophyll precursors [1,2,28]; does ALA enhance low-temperature resistance in tomatoes by affecting chlorophyll-related synthesis and metabolism? This experiment measured the accumulation of iron in the leaves of tomato seedlings. Iron is essential for chlorophyll synthesis, so the iron content of tomato seedlings after exogenous application of ALA was measured in the experiment. In this study, there was no substantial change in iron accumulation in the leaves of tomato seedlings sprayed with or without exogenous ALA at normal temperature (Figure 2). The accumulation of iron in the leaves of LT-treated tomatoes was significantly reduced by 56.30% compared to CK. Notably, the accumulation of iron in tomato leaves treated with exogenous ALA at low temperature (LTA) was significantly increased by 101.15% compared to LT. In addition, this experiment measured the expression of iron transport-related genes under low-temperature stress and the results show that SlZIP1 and SlZIP3 were significantly induced by low temperature, and exogenous ALA further induced the expression of these two genes under low temperature (Figure S2).

**Figure 3:**
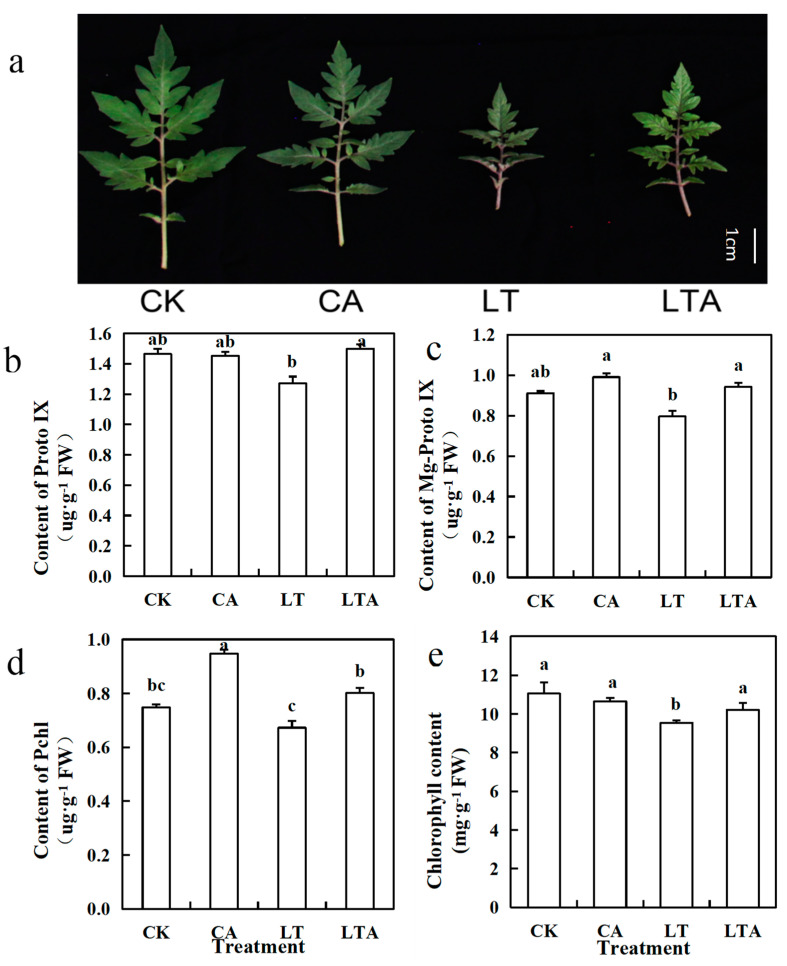
Effects of exogenous ALA on chlorophyll synthesis in tomato seedling leaves under low-temperature stress. Some of the tomato seedlings with five true leaves were grown at normal temperature and pre-sprayed with distilled water or ALA. Some of the tomato seedlings were grown at low temperature and pre-sprayed with distilled water or ALA. After nine days of low-temperature treatment, (**a**) leaf growth condition, the contents of (**b**) Proto IX, (**c**) Mg-proto IX, (**d**) Pchl, and (**e**) chlorophyll were measured. Values represent averages of three independent measurements, and error bars represent standard errors. Different letters above the columns indicate significant difference according to the Tukey test (*p* < 0.05).

The leaf area treated with ALA at low temperature was significantly bigger than that of LT, and the leaf color was brighter and greener. Compared with CK, LT greatly reduced the content of the contents of chlorophyll and its synthetic precursors (Proto IX, Mg-proto IX, and Pchl). Each index decreased by 15.18%, 13.24%, 12.53%, and 10.15%, respectively, compared with CK. However, the above indicators of LTA significantly increased by 10.11%, 17.94%, 18.34%, and 19.17%, respectively, compared with LT (Figure 3b–e), and also increased leaf area (Figure 3a, Figure S5). In summary, it was hypothesized that exogenous ALA could alleviate the inhibitory effect of low temperature on chlorophyll synthesis and increase chlorophyll content, and thus enhance photosynthesis to alleviate low-temperature stress by promoting the synthesis of chlorophyll precursors.

### 2.3. Exogenous ALA Increased ABA Content in Tomato Seedlings Leaves under Low Temperature

**Figure 4:**
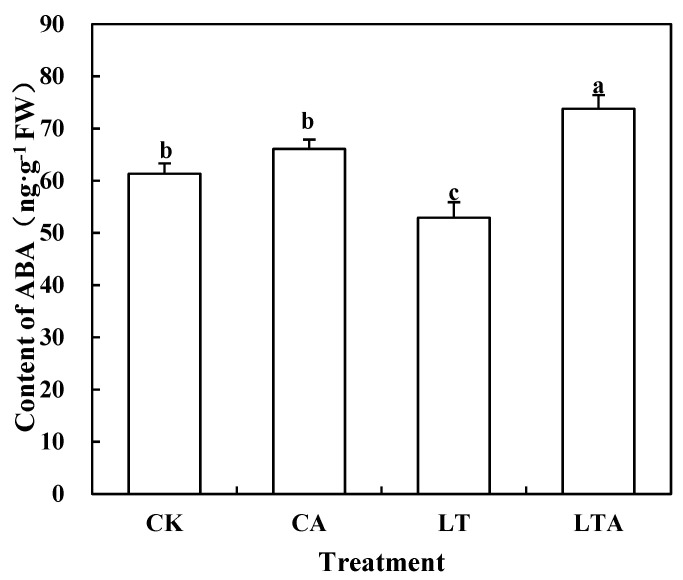
Effects of exogenous ALA on ABA content in tomato seedlings leaves. Some of the tomato seedlings with five true leaves were grown at normal temperature and pre-sprayed with distilled water or ALA. Some of the tomato seedlings were grown at low temperature and pre-sprayed with distilled water or ALA. The ABA content of tomato seedling leaves treated for nine days was measured at different temperatures. Values represent averages of three independent measurements, and error bars represent standard errors. Different letters above the columns indicate significant difference according to the Tukey test (*p* < 0.05).

**Figure 5:**
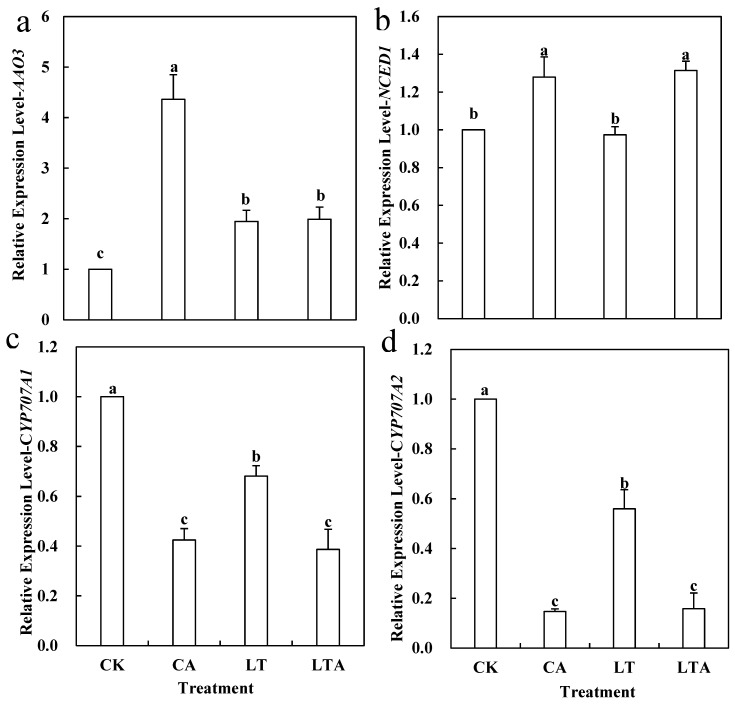
Effect of exogenous ALA on the expression of ABA metabolism-related genes. (**a**) *AAO3* and (**b**) *NCED1* are ABA synthesis-related genes; (**c**) *CYP707A1* and (**d**) *CYP707A2* are ABA degradation-related genes. Some of the tomato seedlings with five true leaves were grown at normal temperature and pre-sprayed with distilled water or ALA. Some of the tomato seedlings were grown at low temperature and pre-sprayed with distilled water or ALA. After 24 h of low-temperature treatment, the expression levels of *AAO3*, *NCED1*, *CYP707A1*, and *CYP707A2* in the leaves were measured. Values represent averages of three independent measurements, and error bars represent standard errors. Different letters above the columns indicate significant difference according to the Tukey test (*p* < 0.05).

ABA, as a stress hormone, plays an important role in alleviating abiotic stress in plants. Previous studies showed that ABA can affect chlorophyll metabolism under adversity stress to take part in stress regulation [24]; does there exist a functional link between ALA and ABA? Under low temperature, the ABA content in tomato leaves decreased by 13.74%, while spraying exogenous ALA (LTA) increased ABA content by 39.43% (Figure 4). Furthermore, this experiment measured ABA content in leaves at several time points during the early stages of treatment (Figure S3), and the data show that spraying exogenous ALA promoted the accumulation of ABA under low-temperature stress. Further, we measured the expression of ABA synthesis and degradation-related genes *AAO3*, *NCED1* and *CYP707A1*, and *CYP707A2* after ALA treatment (Figure 5). The application of ALA at room temperature promoted the expression of *AAO3* and *NCED1* and inhibited the expression of *CYP707A1* and *CYP707A2*, but the expression of *AAO3* did not change significantly after low-temperature treatment, *NCED1* expression increased significantly, and the expression of *CYP707A1* and *CYP707A2* was significantly inhibited (Figure 5). It is suggested that exogenous ALA may inhibit ABA degradation mainly by suppressing the expression of ABA degradation genes, and thus promoting ABA accumulation at low temperature.

### 2.4. Exogenous ABA Promotes the Accumulation of Iron, Chlorophyll and Its Precursors, and Improves the PS II Maximum Photochemical Efficiency of Plants under Low-Temperature Stress

**Figure 6:**
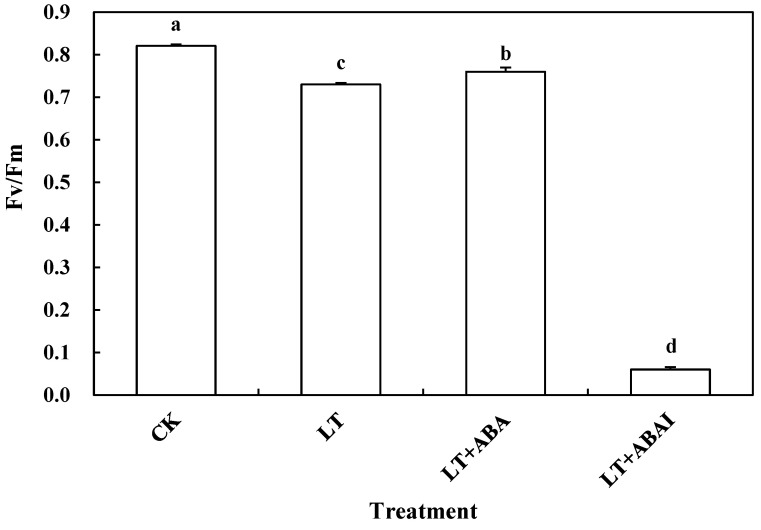
Effect of exogenous ABA on Fv/Fm of tomato seedlings under low temperature. Tomato seedlings with five true leaves were sprayed with distilled water, ABA, or fluridone (LT+ABAI), placed at normal temperature, and protected from light for 12 h before starting the low-temperature treatment. CK was kept at normal temperature and pre-sprayed with distilled water. After nine days of low-temperature treatment, Fv/Fm of the plant leaves were measured. Values represent averages of three independent measurements, and error bars represent standard errors. Different letters above the columns indicate significant difference according to the Tukey test (*p* < 0.05).

Photosynthetic performance is one of the indicators of plant stress tolerance, and ALA can improve chlorophyll accumulation, enhance photosynthesis, and thus improve cold tolerance. Additionally, the accumulation of ABA in plants increased after external application of ALA; can ABA and ALA function similarly enhance photosynthetic performance to improve plant cold tolerance? This experiment measured the Fv/Fm of tomato seedlings after the application of ABA and ABA inhibitors. Compared with CK, the Fv/Fm of leaves treated with LT was significantly reduced by 8.37%, yet this parameter of LT+ABA leaves significantly increased by 8.45% compared with LT. It is worth noting that the Fv/Fm of leaves treated with fluridone (LT+ABAI) under low-temperature stress was significantly reduced by 91.78% compared to LT (Figure 6). Thus, exogenous ABA plays an important role in maintaining the photosynthetic characteristics of tomato seedling leaves under low-temperature stress.

**Figure 7:**
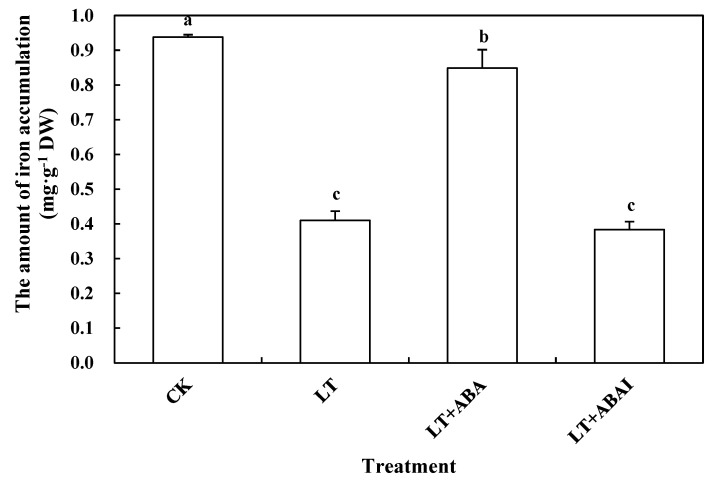
Effects of exogenous ABA on iron accumulation in tomato seedling leaves. Tomato seedlings with five true leaves were sprayed with distilled water, ABA, or fluridone (LT+ABAI), placed at normal temperature, and protected from light for 12 h before starting the low-temperature treatment. CK was kept at normal temperature and pre-sprayed with distilled water. Iron accumulation was measured at different temperatures for nine days. Values represent averages of three independent measurements, and error bars represent standard errors. Different letters above the columns indicate significant difference according to the Tukey test (*p* < 0.05).

The accumulation of ABA was increased by exogenous ALA at low temperature, so this experiment measured the accumulation of iron, chlorophyll, and its precursors to investigate whether ABA played a similar role as ALA under low temperature. The iron accumulation of LT was significantly reduced by 56.01% compared with CK (Figure 7). Under low-temperature stress, spraying exogenous ABA significantly increased the iron accumulation in leaves by 97.56%, whereas inhibition of ABA synthesis (LT+ABAI) did not significantly decrease iron content (Figure 7). Additionally, under low-temperature stress, exogenous ABA significantly induced the expression of transport-related genes (SlZIP1 and SlZIP3) (Figure S2).

**Figure 8:**
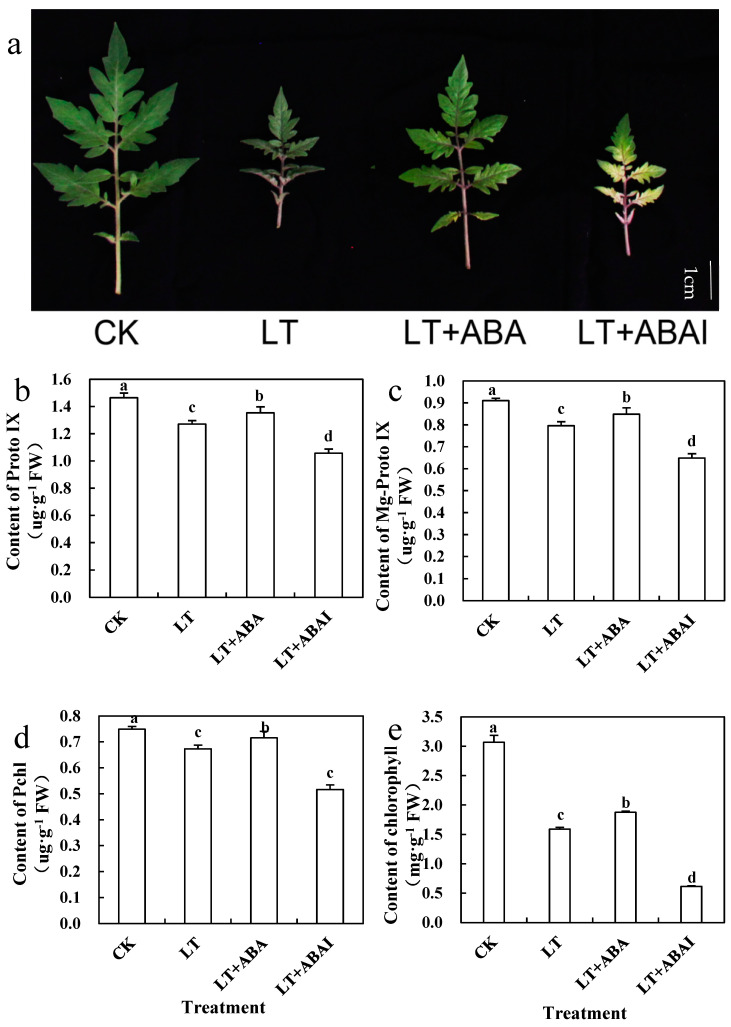
Effects of exogenous ABA on chlorophyll synthesis in tomato seedling leaves under low-temperature stress. Tomato seedlings with five true leaves were sprayed with distilled water, ABA, or fluridone (LT+ABAI), placed at normal temperature, and protected from light for 12 h before starting the low-temperature treatment. CK was kept at normal temperature pre-sprayed with distilled water. After nine days of low-temperature treatment, (**a**) leaf growth condition, the contents of (**b**) Proto IX, (**c**) Mg-proto IX, (**d**) Pchl, and (**e**) chlorophyll were measured. Values represent averages of three independent measurements, and error bars represent standard errors. Different letters above the columns indicate significant difference according to the Tukey test (*p* < 0.05).

Low-temperature stress can lead to severe impairment of photosynthesis in tomato, while persistent low temperatures can also lead to loss of green color in tomato leaves [2,3]. Leaf areas and the spread of tomato seedlings treated with ABA at low temperature were significantly better than that of control, and the color of the tomato seedling leaves was brighter and greener (Figure 8a, Figure S6). The content of chlorophyll, Proto IX, Mg-proto IX, and Pchl in LT decreased by 47.42%, 13.24%, 12.53%, and 7.65%, respectively, compared to CK. However, the corresponding indexes of LT+ABA increased by 15.89%, 6.45%, 6.66%, and 6.24%, respectively, compared to LT (Figure 8b–e). In addition, to verify whether the effect was produced by ABA, we sprayed fluridone under low-temperature stress and the results show that spraying fluridone (LT+ABAI) under low-temperature stress eliminated the effect of ABA and significantly inhibited chlorophyll synthesis, which resulted in a significant decrease of 60.61% in chlorophyll content of LT+ABAI. The above results show that exogenous ABA could alleviate the inhibitory effect of low temperature on chlorophyll synthesis and increase chlorophyll content by promoting the synthesis of chlorophyll precursors.

### 2.5. Exogenous ABA Induces the Expression of Proto IX Key Enzyme for ALA Synthesis and Reduces the Accumulation of ALA

**Figure 9:**
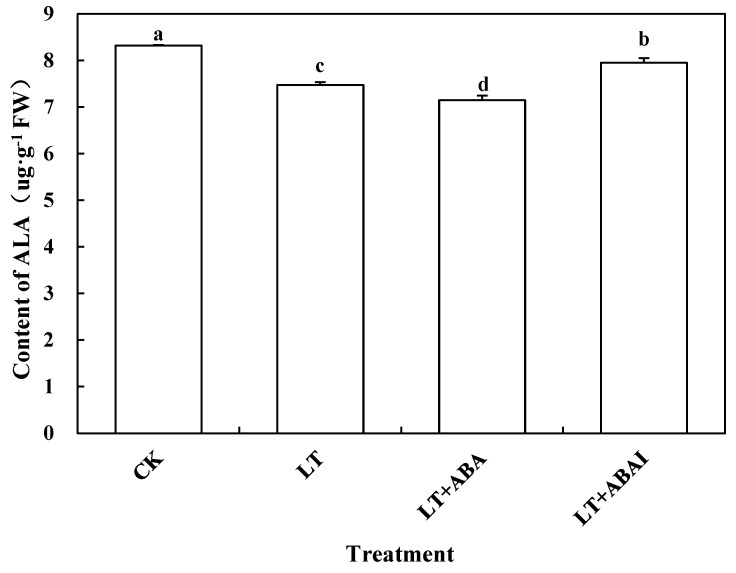
Effects of exogenous ABA on ALA content in tomato seedling leaves. Tomato seedlings with five true leaves were sprayed with distilled water, ABA, or fluridone (LT+ABAI), placed at normal temperature, and protected from light for 12 h before starting the low-temperature treatment. CK was kept at normal temperature and pre-sprayed with distilled water. The ALA content of tomato seedling leaves treated for nine days was measured at different temperatures. Values represent averages of three independent measurements, and error bars represent standard errors. Different letters above the columns indicate significant difference according to the Tukey test (*p* < 0.05).

**Figure 10:**
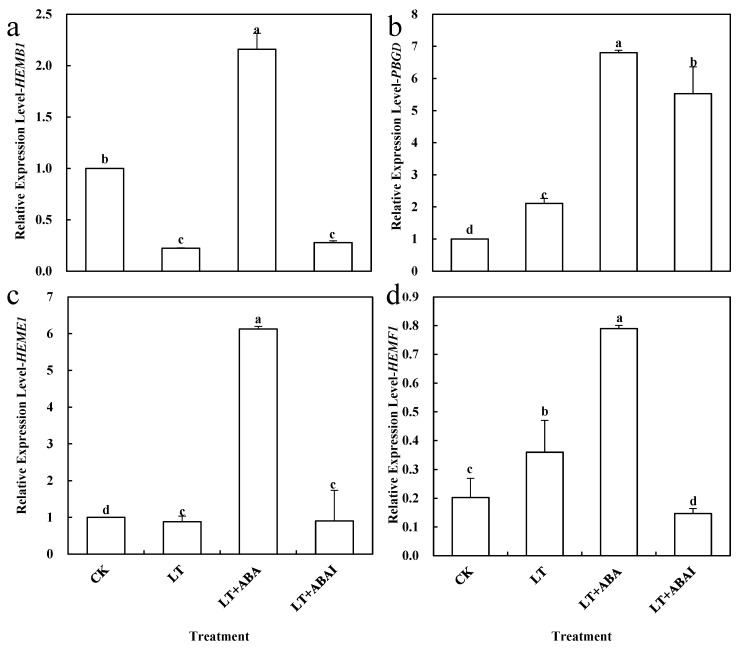
Effects of ABA on Proto IX synthesis. Relative mRNA abundance of (**a**) *HEMB1*, (**b**) PBGD, (**c**) *HEME1*, and (**d**) *HEMF1*. Tomato seedlings with five true leaves were sprayed with distilled water (LT), 100 μM ABA (LT+ABA), or 100 μM fluridone (LT+ABAI), placed at normal temperature, and protected from light for 12 h before starting the low-temperature treatment (8–18 °C/8–12 °C, day/night). CK was kept at normal temperature (16–28 °C/16–18 °C, day/night) pre-sprayed with distilled water. After 24 h of low-temperature treatment, the expression levels of *HEMB1*, *PBGD*, *HEME1*, and *HEMF1* in the leaves were measured. Values represent averages of three independent measurements, and error bars represent standard errors. Different letters above the columns indicate significant difference according to the Tukey test (*p* < 0.05).

**Figure 11:**
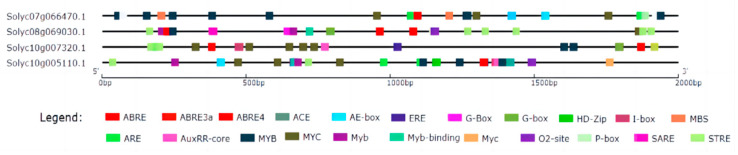
Analysis of cis-elements in the promoters of Proto IX synthesis-related genes in tomato. Each colored block represents functionally similar promoter cis-elements. The four genes shown are *SlPBGD*, *SlHEMB1*, *SlHEME1,* and *SlHEMF1*.

The content of ALA in the leaves of LT+ABA was significantly reduced by 5.75% compared with LT, while the ALA content of LT+ABAI was significantly increased by 13.48% compared with LT (Figure 9). This shows that the external application of ABA under low-temperature stress caused a significant decrease in ALA content, while the application of fluridone promoted ALA accumulation. *HEMB1*, *PPGD*, *HEME1,* and *HEMF1* are key enzymes in the synthesis of Proto IX by ALA. To further investigate the relationship between ABA and ALA, this experiment measured the changes in the expression of the above genes after 24 h of low-temperature stress. The expression levels of *HEMB1*, *PPGD*, *HEME1*, and *HEMF1* were significantly induced by ABA under low-temperature stress (Figure 10). To confirm this result, we performed promoter analysis of all the various genes mentioned above that are up-regulated by ABA, and the results show that all of these genes contain multiple ABA response-related cis-acting elements ABRE. This further suggests that ABA may be involved in the ALA metabolic pathway (Figure 11). Thus, ABA may be involved in the synthesis of Proto IX from ALA by inducing the expression of *HEMB1*, *PPGD*, *HEME1*, and *HEMF1*.

## 3. Discussion

Studies show that ALA is not only a metabolic intermediate in plants, but can also be applied as a growth regulator similar to ABA in regulating plant adversity stress. Balestrasse et al. found that treatment of soybeans with low concentrations of ALA at low temperatures reduced cellular damage from reactive oxygen species, improved photosynthetic efficiency, and mitigated the adverse effects of low temperatures [29]. Korlcmaz et al. showed that pepper treated with exogenous ALA maintained the antioxidant enzyme activity, increased the content of osmoregulatory substances, and increased the cold resistance of the plant [30]. This study found that under low-temperature stress, Fv/Fm, relative conductivity h, and proline content of tomato seedling leaves sprayed with exogenous ALA significantly increased or decreased by 5.31%, 6.20%, and 25.48%, respectively, compared with CK, while plant growth status was also significantly better than CK (Figure 1), indicating that exogenous ALA application could indeed alleviate low-temperature stress in tomato seedlings.

The effect of ALA on plants under adversity stress is more similar to that of ABA and both can alter plant resistance to abiotic stresses by regulating osmoregulatory substances, a reactive oxygen species scavenging system, and photosynthesis [31]. The low-temperature treatment resulted in a significant decrease in chlorophyll content by 15.18% compared to CK (Figure 3). In the present study, chlorophyll content was found to be significantly up-regulated after external application of ALA under low-temperature stress. Similarly, ABA can also increase the chlorophyll content of plant leaves under adversity stress [32,33]. Based on the similar effects of ALA and ABA in previous studies, their effects on iron accumulation and chlorophyll synthesis under low-temperature stress were investigated in this experiment. The results show that the maximum photochemical efficiencies of iron, chlorophyll, and PS II were significantly increased by 101.15%, 10.11%, 5.31%, and 97.56%, 15.89%, and 8.45% after exogenous ALA and exogenous ABA treatments, respectively, under low-temperature stress (Figure 2, Figure 3, Figure 6 and Figure 7). The functions of both are similar to previous studies and suggest a possible interrelationship between ALA and ABA [2,32,33,34]. Yan found that exogenous ALA could enhance ABA accumulation in cucumber seedlings under salt stress [35]. Wang et al. found that exogenous ALA promoted ABA accumulation in maize seedlings as the duration of low-temperature treatment increased [36]. Cao et al. found that ABA signaling mediated 5-aminolevulinic acid-induced anthocyanin biosynthesis in red pear fruits [37]. So does ALA have a similar effect on ABA in tomatoes at low temperature? This experiment measured the ABA content after low temperature external application of ALA and found a significant increase of 39.43% (Figure 4); meanwhile, qRT-PCR results show that ALA could significantly regulate the expression of ABA metabolic genes (Figure 5). These results suggest that ALA can promote the accumulation of ABA at low temperature.

Additionally, this experiment explored whether ABA could also have an effect on ALA. Interestingly, inhibition of ABA increased the amount of ALA, but the leaves of tomato seedlings became significantly yellow and the amount of chlorophyll and Proto IX did not increase (Figure 8). QRT-PCR also revealed that the expression of several key enzyme genes involved in chlorophyll synthesis by ALA, *HEMB1*, *PPGD*, *HEME1,* and *HEMF1* was significantly up-regulated (Figure 10). However, these genes showed different trends under low temperature and ABAI treatment, which may be due to their different roles in chlorophyll synthesis, and their specific mechanisms of action need to be further investigated. In general, chlorophyll synthesis is inhibited under low-temperature stress, whereas exogenous application of ABA promoted chlorophyll synthesis. It is hypothesized that the key enzyme of the ALA synthesis chlorophyll prerequisite can be activated by the ABA signaling pathway under low-temperature stress, which eventually regulates chlorophyll synthesis. In contrast, exogenous application of ABA led to a decrease in plant ALA content because it accelerated the process of ALA synthesis to Proto IX. This may be one of the mechanisms to alleviate stress by regulating photosynthesis under phytoplanktonic adversity conditions, but it is only a preliminary conjecture at present, and its specific mechanism of action needs to be verified by further experiments. However, what are the specific inter-regulatory processes between ABA and ALA under abiotic stress and which key genes are involved in them are still questions that deserve further study.

**Figure 12:**
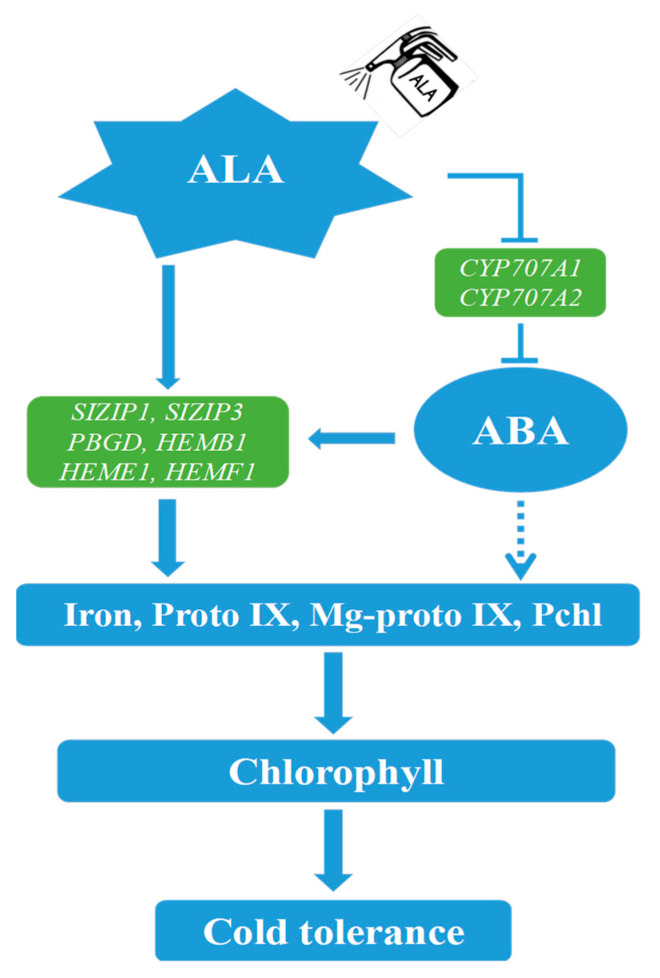
A model of ALA and ABA crosstalk to enhance cold tolerance in tomato seedlings. Under low-temperature stress, exogenous ALA could, on the one hand, increase the content of Pchl and iron by inducing the expression of ProtoIX synthesis-related genes (*SlPBGD*, *SlHEMB1*, *SlHEME1*, and *SlHEMF1*) and iron utilization-related genes (*SlZIP1*, *SlZIP3*), and thus promote chlorophyll synthesis; on the other hand, exogenous ALA could promote the accumulation of ABA by inhibiting the expression of ABA degradation-related genes (*SlCYP707A1*, *SlCYP707A2*), thus further increasing the content of Pchl and iron. It is worth mentioning that ABA can also participate in the synthesis of Proto IX from ALA by inducing the expression of key enzyme genes of chlorophyll precursors (*SlPBGD*, *SlHEMB1*, *SlHEME1*, and *SlHEMF1*). In conclusion, ABA may crosstalk with ALA to improve the cold tolerance of tomatoes by regulating iron accumulation and chlorophyll synthesis.

Iron is essential for chlorophyll synthesis, which protects the chloroplast structure from disintegration. Low temperature can limit the ability of plant roots to absorb Fe^2+^ and lead to leaf shrinkage [12]. This study found that low-temperature stress resulted in a 56.30% decrease in iron accumulation in tomato seedling leaves, while ABA and ALA were able to increase chlorophyll content to alleviate low-temperature stress, and further measurements revealed that both ALA and ABA increased iron accumulation under low temperature. Interestingly, under low-temperature stress, although ABA was able to promote iron accumulation, iron accumulation in leaves did not decrease substantially after ABA synthesis was inhibited (Figure 7). To initially investigate the regulatory principles, we measured the expression of *SlZIP1* and *SlZIP3*, key genes involved in iron transport, after external application of ALA and ABA at low temperature and found that they were significantly up-regulated (Figure S2) [38]. Therefore, we speculated that it is because inhibition of ABA synthesis leads to an increase in ALA content, which can also promote the expression of iron transporter-related genes and iron accumulation, thus counteracting the negative effects of the ABA synthesis inhibitor. Although, the external application of ABA reduced ALA but still increased iron accumulation to a large extent, which might be related to the ABA concentration and the degree of ALA reduction in accumulation. In summary, ABA may be involved in ALA-regulated iron accumulation and chlorophyll synthesis in tomato seedling leaves under low-temperature stress (Figure 12), but the specific mechanism of action and the interrelationship between them need further studies.

## 4. Materials and Methods

### 4.1. Plant Material and Growth Conditions

The cold-sensitive tomato cultivar ‘Jin Peng No.1′ was cultivated as described in our previous report [1,2]. A 3:1:1 mixture of grass charcoal, vermiculite, and perlite was used as a substrate for each tomato material. Seedlings with completely expanded fourth true leaves were transplanted in nutrition pots (7 cm × 7 cm), one plant per nutrition pot. Subsequently, they were left to continue growing at normal temperature (16–28 °C/16–18 °C, day/night, Figure S1), relative humidity of 65% ± 5%, and a photoperiod of 10.5 h [photosynthetic photon flux density (PPFD), 350 μmol·m^−2^s^−1^] + 1.5 h (PPFD, 50 μmol·m^−2^s^−1^)/12 h (day/night).

When the fifth true leaves completely expanded, plants of similar appearance were selected for the experiments. A total of six treatments were performed in this experiment (Table 1). According to the screening of our group’s previous research, the concentration of exogenous ALA used in this study was 25 mg·L^−1^ [1,2]. Exogenous ALA (25 mg·L^−1^), ABA (100 µM), and fluridone (an ABA inhibitor, 100 µM), respectively, containing 0.02% Silwet L-77 were separately sprayed 12 h before low temperature, and the application rate of these solutions was 6 mL/plant [24,39,40]. The above reagents were purchased from Sigma Aldrich (St. Louis, MO, USA). After 12 h of dark cultivation (16–18 °C), all seedlings, except for the control group grown at normal temperature (the temperature is the same as the above four-leaf culture period, Figure S1), were subjected to low-temperature treatment (8–18 °C/8–12 °C, day/night, Figure S1) for 9 days while keeping other environmental conditions, such as photoperiod (12/12 h) and humidity (60–70%). The first day of treatment at 9:00 a.m. was used as the sampling time of 0 h. Fully expanded third functional leaves treated for 24 h were collected to measure gene expression; leaves treated for 6, 12, 24 h, and 3 or 9 days were collected to measure ABA content; and leaves treated for 9 days were collected to measure the content of iron, chlorophyll, and their precursors. Fifteen plants were analyzed for each treatment in one replicate. Three independent biological experiments, which had three technical repetitions, were performed to ensure the reproducibility of results.

### 4.2. Chlorophyll Fluorescence Parameter’s

Fv/Fm was measured according to the methods of Pérez-Bueno et al. with the Open FluorCam FC 800-O and analyzed using the Fluorcam7 software FC800 (PSI, Brno, Czech Republic) [41].

### 4.3. Determination of Relative Conductivity and Proline Contents

The relative conductivities of leaves of different treatments were calculated according to the method described by Tan et al.; ground 0.1 g frozen samples of leaf tissue [42]. Approximately 0.1 g of frozen leaf tissue samples were thoroughly ground with a 3% sulfosalicylic acid solution and free proline content was determined according to the method described by Chouj et al. [43].

### 4.4. Extraction and Determination of ABA

ABA was extracted from tomato seedling leaves following the method of Müller and Munné-Bosch with slight modifications [44]. For ABA extraction, 0.2 g of leaves were used. The leaves were frozen in liquid nitrogen, powdered with high-throughput tissue lyser (Scienz, Ningbo, China), and extracted with methanol, isopropanol, and acetic acid (20:79:1 [*v*/*v*]) at 4 °C in the dark overnight. The samples were then vortexed and centrifuged at 14,000× *g* 4 °C for 5 min. The supernatants were filtered through a 0.45 μm disposable needle filter. The samples were then analyzed via liquid chromatography–mass spectrometry (QTRAP 5500, AB SCIEX, Framingham, MA, USA). Quantification was conducted by the standard addition method by spiking control plant samples with ABA solutions (ranging from 5 ng·mL^−1^ to 100 ng·mL^−1^).

### 4.5. Evaluation of Iron Accumulation Amount

Plant leaves were cleaned with distilled water, enzymes were deactivated at 105 °C for 15 min, and the leaves were dried to a constant weight at 75 °C until the dry matter was obtained. The dry plant samples were crushed and sieved (0.5 mm) with a micro crusher. These samples were boiled in HNO_3_ and H_2_O_2_. Afterward, the iron content was determined as follows by atomic absorption spectrometry with an atomic absorption spectrophotometer (Z-5000, Perkin Elmer Enterprise Management Co., Ltd., Waltham, MA, USA) [45].

### 4.6. Evaluation of Chlorophyll and Its Precursors Contents

Total chlorophyll (Chl) contents were measured following the method of Goodwin [46]. Protoporphyrin IX (Proto IX), Mg-protoporphyrin (Mg-proto IX), and protochlorophyllide (Pchl) contents were measured according to the method of Arnon [47]. ALA content was determined using the plant 5-aminolevulinic acid enzyme-linked immunosorbent assay kit (Shanghai FANKEL Industrial Co., Fankew brand, F7958, Shanghai, China) following the manufacturer’s instructions.

### 4.7. RNA Extraction and Gene Expression Analyses

The relative expression of genes related to iron transport-related genes zinc–iron transporter protein 1, 3 (SIZIP1, SIZIP3), ABA synthesis and degradation-related genes aldehyde oxidase, beta-carotene dioxygenase, abscisic acid 8’-hydroxylase 1, abscisic acid 8’-hydroxylase 2 (*AAO3*, *NCED1*, *CYP707A1*, and *CYP707A2*), and that of key genes involved in the ALA biosynthetic pathway, including porphobilinogen synthase, hydroxymethylbilane synthase, homocysteine methyltransferase, coproporphyrinogen oxidase (HEMB1, PBGD, HEME1, and HEMF), were measured via real-time quantitative PCR (RT-qPCR) [23,48]. Total RNA was extracted using a plant RNA extraction kit (OmegaBio-Tek, Doraville, GA, USA) according to the manufacturer’s recommendations. Reverse transcription was performed using a PrimeScript TM RT reagent kit (Takara, Shiga, Japan) following the manufacturer’s instructions. For RT-qPCR, Takara TB GreenT Premix Ex Taq″m II (Takara, Shiga, Japan) in 20 μ reactions was used using an ABI StepOne Plus Real-Time PCR System (Applied Biosystems, Carlsbad, CA, USA). Relative levels of gene expression were calculated as described by Livak and Schmittgen [49]. Three biological replicates were performed for each experiment. Table S1 shows the primers of *Actin7*, *SIZIP1*, *SIZIP3*, *AAO3*, *NCED1*, *CYP707A1*, *CYP707A2*, HEMB1, PBGD, HEME1, and HEMF1.

### 4.8. Promoter Element Analyses of SlPBGD, SlHEMB1, SlHEME1 and SlHEMF1

The 2000 bp upstream sequences of SlPBGD, SlHEMB1, SlHEME1, and SlHEMF1 were from the Phytozome database. The cis-acting elements were searched by PlantCARE online software (http://bioinformatics.psb.ugent.be/webtools/plantcare/html/, accessed on 14 May 2023) and visualized by TBTools.

### 4.9. Statistical Analyses

Data were analyzed with SPSS 20 statistical software (IBM SPSS statistics 20.0, Armonk, NY, USA) with Tukey’s multiple range test (*p* < 0.05). Each reported data point represented the average of three independent experiments and was presented as mean ± SD.

## 5. Conclusions

Under low-temperature stress, Exogenous ABA and ALA promoted iron uptake, increased the content of Proto IX, Mg-Proto IX, and Pchl, and finally promoted chlorophyll synthesis in leaves. The synthesis of chlorophyll improved the photochemical efficiency, thus further improving the tolerance of tomato under low-temperature stress. In this biochemical process, Exogenous ALA promoted the accumulation of ABA. Meanwhile, ABA may be involved in the synthesis of chlorophyll from ALA, predicting that there may be a close interrelationship between ALA and ABA. ABA crosstalk with ALA improved tomato low-temperature stress tolerance by regulating chlorophyll synthesis and iron accumulation. It is worth mentioning that this study only preliminarily analyzed the possible crosstalk relationship between ALA and ABA under low-temperature stress. Whether they have different interactions under different adversity, and the specific mechanism between them, needs to be further explored by means of more molecular research.

## Figures and Tables

**Table 1:** Experimental treatments.

Number	Treatment
CK	normal temperature + distilled water
CA	normal temperature + 25 mg·L^−1^ ALA
LT	low-temperature + distilled water
LTA	low-temperature + 25 mg·L^−1^ ALA
LT+ABA	low-temperature + 100 µM ABA
LT+ABAI	low-temperature + 100 µM fluridone

## Data Availability

All data generated or analysed during this study are included in this published article [and its Supplementary Materials].

## Supplementary Materials

The supporting information can be downloaded at: https://www.mdpi.com/article/10.3390/ijms241310781/s1.

## Abbreviations

ABA, abscisic acid; ALA, 5-aminolevulinic acid; Chl, chlorophyll; Mg-proto IX, Mg-protoporphyrin IX; Pchl, protochlorophyll; Proto IX, protoporphyrin IX; PS II, photosynthetic system II; qRT-PCR, quantitative real-time PCR; ROS, reactive oxygen species; MDA, malondialdehyde.
