# Supplementary material for: Crosstalk between 5-Aminolevulinic Acid and Abscisic Acid Adjusted Leaf Iron Accumulation and Chlorophyll Synthesis to Enhance the Cold Tolerance in Solanum lycopersicum Seedlings
Source: Int J Mol Sci. 2023 Jun 28;24(13):10781. doi: 10.3390/ijms241310781 (PMC10341924; doi:10.3390/ijms241310781)
Supplement: Supplementary file 1 [file ijms-24-10781-s001.zip › ijms-2444891-supplementary.pdf]

Supplementary data

Table S1. Primers for qRT-PCR.

Gene name	Accession numbers	Gene annotation	Forward primer	Reverse primer
SlActin	Solyc11g065990	actin	GGGATGGAGAAGTTTGGTGGTGG	CTTCGACCAAGGGATGGTGTAGC
SlPBGD	Solyc07g066470	hydroxymethylbilane synthase / uroporphyrinogen synthetase	TTCTTCGCCTTGCCGTAAAT	CTTCCTCGTGTTCCAACTCTGAT
SlHEMB1	Solyc08g069030	porphobilinogen synthase / delta-aminolevulinic acid dehydratase	CCTTTGCCCATAGCTGCCTA	GCGATGTTTTCTCGCAGTGG
SlHEME1	Solyc10g007320	homocysteine methyltransferase related	TGTGAAAAAGGGGAAAGTGGGT	AAGGAGAGCAGAGGGAGTCT
SlHEMF1	Solyc10g005110	coproporphyrinogen oxidase	TACAGTTGGTGCAAAGGGGT	TGTCACTGAACCACTAGCCA
SlZIP1	Solyc11g062020	zinc–iron transporter protein 1	GGCTCATAATGCTATAAACTCC	GGCACTTGAAATGGGTG
SlZIP3	Solyc01g087530	zinc–iron transporter protein 3	GAGTCATCCTCCCAATC	TGTATAAACCCTGTGGC
SlAAO3	Solyc11g071580	Aldehyde oxidase / Retinal oxidase	TCTGACTGGAGAGACTGCCA	ACGACAAAGGGAAGAACCTGT
SlNCED1	Solyc07g056570	Beta-carotene dioxygenase / 9-cis-epoxycarotenoid dioxygenase *NCED3*	TGTTGCAAACGCCGGTTTAG	GCCGGTGGGTGTTACCTTTA
SlCYP707A1	Solyc04g078900	abscisic acid 8'-hydroxylase / ABA 8'-hydroxylase	TGGCAGTGGTGTACATGCTT	CCAATGGGACTGGGAATGGT
SlCYP707A2	Solyc08g005610	Abscisic acid 8'-hydroxylase 1-related	TCTCCCCGGTACACTCTTCA	GAATGACCCGAGCAAATCGC

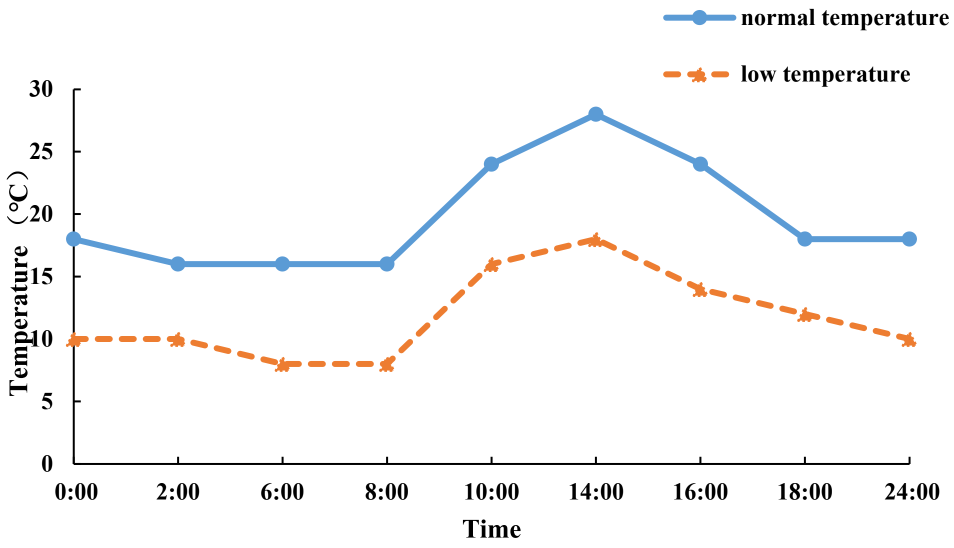

Figure S1. Temperature change in a day during the test.

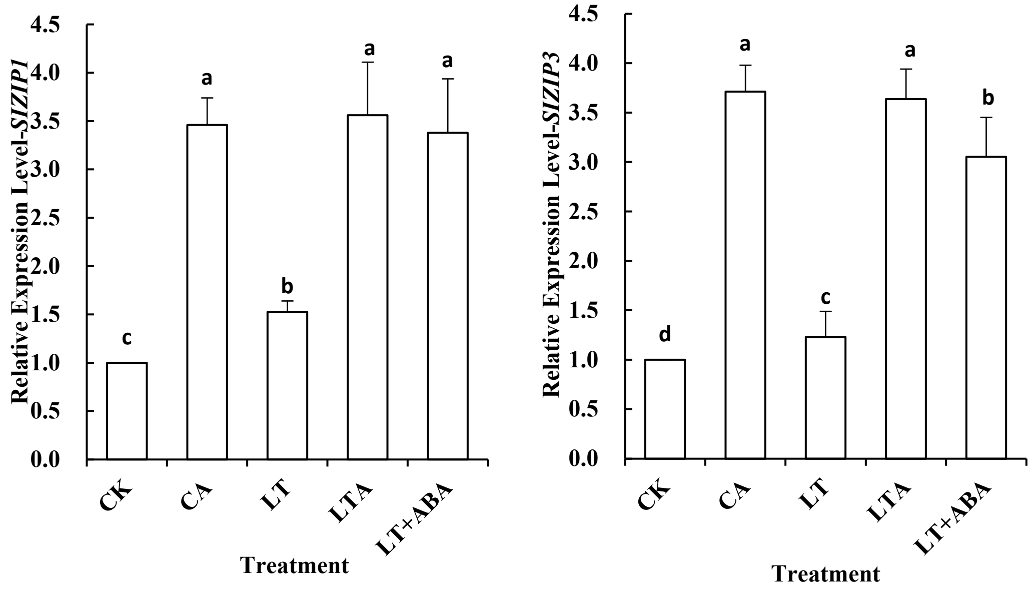

Figure S2. Effects of exogenous ALA and ABA on the expression levels of genes related to zinc–iron transporter.

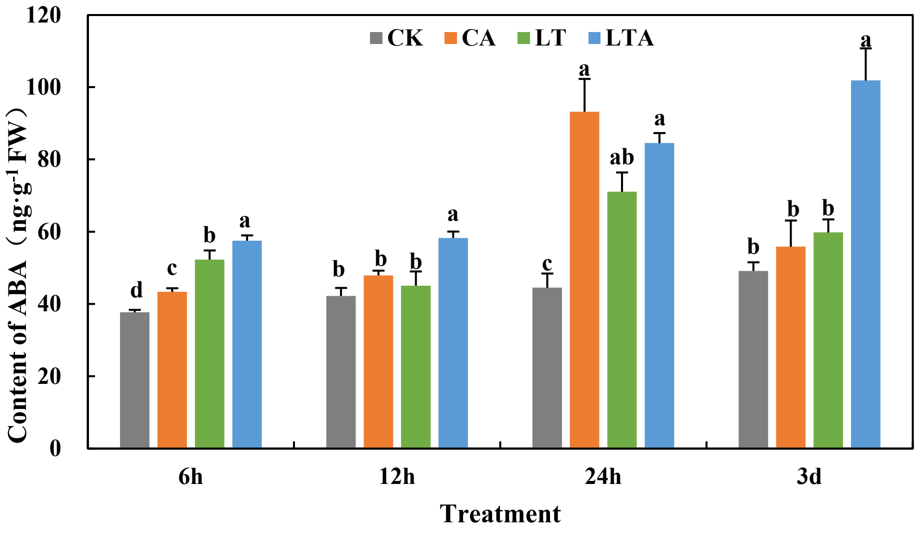

Figure S3. Effects of exogenous ALA on ABA content in tomato seedlings leaves.

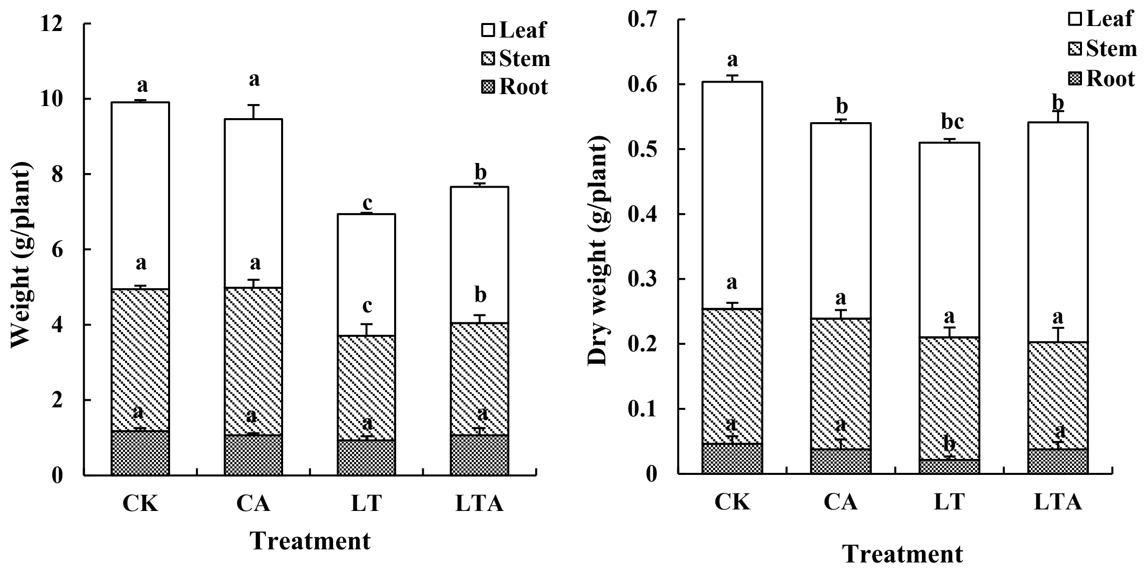

Figure S4. Effect of exogenous ALA on biomass accumulation of tomato seedlings.

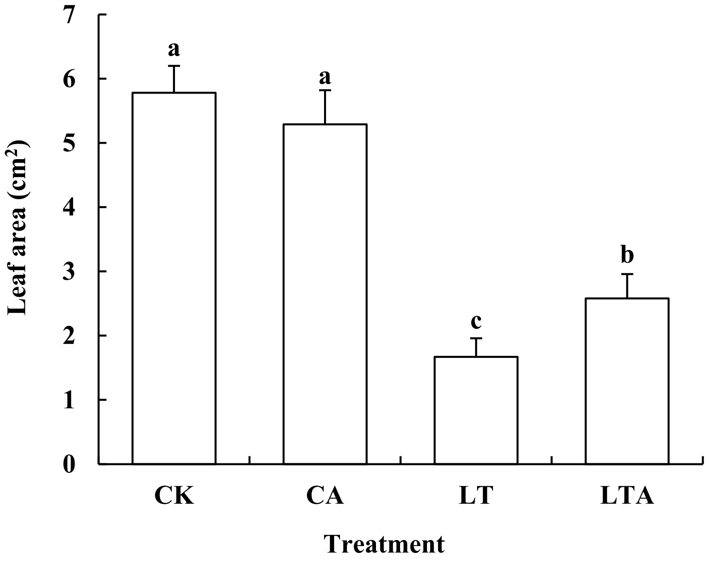

Figure S5. Effects of exogenous ALA on leaf area of tomato seedlings under low temperature stress.

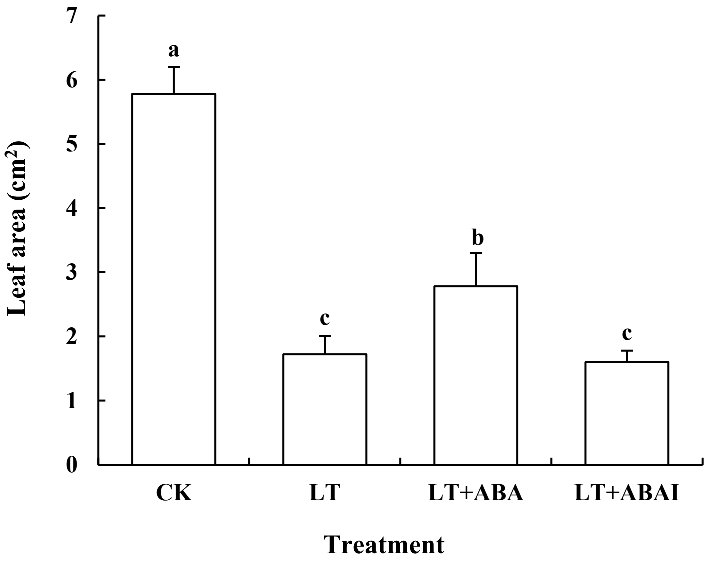

Figure S6. Effects of exogenous ABA on leaf area of tomato seedlings under low temperature stress.
